# Splenic transcriptomic profiling reveals innate immune responses and RIG-I-like receptor signaling in goslings infected with goose astrovirus

**DOI:** 10.3389/fvets.2026.1911277

**Published:** 2026-08-26

**Authors:** Guangzhong Peng, Xinran Guo, Yingjie Gu, Suyu Fan, Xuming Hu, Yang Zhang, Guohong Chen, Wenming Zhao, Qi Xu

**Affiliations:** 1 Jiangsu Key Laboratory for Animal Genetic, Breeding and Molecular Design, College of Animal Science and Technology, Yangzhou University, Yangzhou, China; 2 Institute of Epigenetics and Epigenomics, College of Animal Science and Technology, Yangzhou University, Yangzhou, China

**Keywords:** goose astrovirus, innate immunity, RIG-I-like receptor signaling pathway, spleen, transcriptome

## Abstract

Goose astrovirus (GAstV) is an important pathogen associated with visceral gout, growth retardation, and mortality in goslings, whereas the splenic response to infection remains poorly understood. In this study, GAstV-infected goslings showed positive viral RNA detection in spleen tissues, obvious splenomegaly, and histopathological lesions. Transcriptomic analysis identified 383 differentially expressed genes, including 243 upregulated and 140 downregulated genes. Functional enrichment analyses showed that these genes were mainly involved in antiviral defense, innate immune responses, complement and coagulation cascades, and the RIG-I-like receptor signaling pathway. GSEA further supported the enrichment of the RIG-I-like receptor signaling pathway in infected spleens. Notably, several genes related to this pathway, including *DDX58*, *IFIH1*, *TRIM25*, and *IRF3*, were markedly upregulated and were also identified as hub genes in the protein–protein interaction network. RT-qPCR validation further confirmed the upregulation of these hub genes in GAstV-infected spleens, corroborating the transcriptomic findings. Collectively, these findings indicate that GAstV infection is associated with splenic lesions and extensive transcriptional reprogramming in goslings, and suggest that RIG-I-like receptor-related antiviral signaling may be involved in the splenic innate immune response to GAstV infection. This study provides new transcriptomic insight into the innate immune responses associated with GAstV infection.

## 1 Introduction

Goose astrovirus (GAstV) is an emerging viral pathogen that has caused substantial economic losses in the goose industry in recent years (1, 2). Since its initial identification, GAstV infection has been associated with visceral gout, urate deposition, growth retardation, and increased mortality in goslings (3, 4). As a positive-sense single-stranded RNA virus, GAstV mainly affects young geese, spreads rapidly, and can cause severe disease, thereby posing a serious threat to waterfowl production (5, 6). Although the epidemiology, clinical manifestations, and genomic characteristics of GAstV have been increasingly investigated (7–9), the molecular basis of the host response to GAstV infection remains poorly understood.

The spleen is the largest peripheral immune organ in birds and plays a central role in immune surveillance, antigen processing, lymphocyte activation, and systemic immune regulation (10–12). During viral infection, the spleen often undergoes structural and functional changes and serves as an important site for host antiviral responses and immunopathological processes (13, 14). Previous studies have shown that GAstV infection can induce obvious splenic lesions, including lymphocyte degeneration, apoptosis or necrosis, reticular fiber disruption, and altered immune cell composition (15). In addition, GAstV infection activates innate immune signaling in the spleen, including pattern recognition receptors and downstream molecules such as RIG-I, MDA5, TLR3, MAVS, and IRF3, together with increased expression of interferon and antiviral proteins (16, 17). These findings suggest that the spleen is both a target organ of GAstV-induced injury and a key immune organ involved in antiviral defense. Notably, transcriptomic analysis of chicken astrovirus infection has revealed prominent interferon-induced antiviral signatures in the spleen, supporting the value of splenic transcriptome profiling for identifying key antiviral factors (18). However, the genome-wide transcriptional response and regulatory networks of the goose spleen after GAstV infection remain largely unknown. Therefore, spleen transcriptome analysis is needed to systematically identify the key immune-related genes and pathways involved in GAstV infection.

In this study, we established an experimental GAstV infection model in goslings and performed transcriptomic analysis of spleen tissue using RNA-seq. Differential expression, enrichment, and network analyses were subsequently conducted to investigate the transcriptomic changes induced by GAstV infection. We aimed to characterize the splenic transcriptional response to GAstV infection and identify key genes and pathways potentially involved in antiviral immunity, thereby facilitating further studies of goose antiviral immunity and GAstV pathogenesis.

## 2 Materials and methods

### 2.1 Animals and virus

The GAstV-2 isolate JSYC2503 used in this study was previously isolated and identified in our laboratory, and its complete genome sequence has been deposited in GenBank under accession no. PX548819.1. The strain was originally isolated from kidney, spleen, and liver tissues of a gosling with visceral gout in Yancheng, Jiangsu Province, China. Healthy 3-day-old Sanhua goslings were provided by Yangzhou Xiaopeng Agricultural Technology Co., Ltd. (Yangzhou, China).

### 2.2 Animal experiments

All goslings were reared under standard conditions with free access to feed and water. Before the experiment, all goslings were confirmed to be negative for GAstV by RT-PCR and showed no obvious clinical abnormalities. The goslings were randomly assigned to an infected group and a control group (n = 10 per group), with no significant difference in initial body weight between the groups. Following group assignment, the infected and control groups were housed in separate rooms under the same environmental and husbandry conditions throughout the experiment to minimize cross-transmission. The GAstV inoculum was prepared by diluting the filtered virus preparation in sterile phosphate-buffered saline (PBS), and its infectious titer was determined in goose embryo fibroblast (GEF) cells. Goslings in the infected group were inoculated intraperitoneally with 0.4 mL of the GAstV suspension at 10^6.68^ TCID₅₀/mL, corresponding to approximately 1.9 × 10^6^ TCID₅₀ per bird. Goslings in the control group received an equal volume of sterile PBS via the same route. The clinical status of the goslings was monitored daily throughout the experiment. At 10 dpi, all goslings were humanely euthanized by cervical dislocation, and spleen tissues were collected for gross lesion examination, viral detection, hematoxylin and eosin (H&E) staining, and transcriptome sequencing. Five spleen samples were randomly selected from each group for RNA-seq analysis. Some samples were immediately stored at −80 °C until further use.

### 2.3 RT-PCR detection of GAstV in spleen tissues

Total RNA was extracted from spleen tissues using FreeZol Reagent (Vazyme, R711, Nanjing, China) in accordance with the manufacturer’s instructions. Reverse transcription was performed using HiScript III Reverse Transcriptase (Vazyme, R302-01, Nanjing, China) to generate first-strand cDNA. Five spleen samples were randomly selected from each group for GAstV detection by conventional RT-PCR using ORF2-specific primers: GAstV-F (5′-GAAGGTGACCAAGAAGGTTAC-3′) and GAstV-R (5′-GAGTTGCTGTTTGCCCATTTTC-3′).

### 2.4 RNA sequencing

Total RNA extraction from spleen tissues, library construction, and sequencing were performed by Personal Biotechnology Co., Ltd. (Shanghai, China). Briefly, poly(A) + mRNA was enriched from total RNA using Oligo(dT) magnetic beads and fragmented to approximately 300 bp. First-strand cDNA was synthesized using random hexamer primers and reverse transcriptase, followed by second-strand cDNA synthesis. After PCR amplification, the libraries were size-selected, resulting in a final library size of approximately 450 bp. RNA quality was assessed using an Agilent 2,100 Bioanalyzer, and samples with an RNA integrity number (RIN) ≥ 8 were used for library construction. Libraries that passed quality control were sequenced on the Illumina NovaSeq 6,000 platform to generate 150-bp paired-end reads (PE150).

### 2.5 RNA-seq data processing and quality assessment

Raw reads were quality-filtered by removing adapter sequences, reads containing excessive ambiguous bases, and low-quality reads to obtain clean reads for downstream analysis. Sequencing quality metrics, including Q20, Q30, and GC content, were calculated for each sample. Clean reads were then aligned to the goose reference genome (*Anser cygnoides*; Ensembl, GooseV1.0) using HISAT2 (v2.1.0). The total mapping rate and the proportions of uniquely mapped, multiple mapped, and unmapped reads were calculated to evaluate sequencing quality and alignment performance. Based on the gene annotation file, read counts for each gene were generated using HTSeq (v0.9.1). To further assess the reliability and comparability of the RNA-seq data, count data were transformed using the variance stabilizing transformation (VST) function in the DESeq2 package (v1.46.0). The VST-transformed expression values were used only for visualization analyses, including boxplots, sample correlation analysis, and partial least squares discriminant analysis (PLS-DA). These analyses were performed to assess the consistency of expression distributions, within-group reproducibility, and overall differences in expression patterns between groups.

### 2.6 Differential expression analysis

Differential expression analysis was performed using the DESeq2 package in R based on raw read counts from spleen samples collected at 10 dpi from the GAstV-infected and control groups. Multiple testing correction was performed using the Benjamini–Hochberg method. Genes with an adjusted *p* value < 0.05 and an absolute log2 fold change > 1 were defined as differentially expressed genes (DEGs).

### 2.7 GO and KEGG enrichment analyses

Gene Ontology (GO) enrichment analysis and Kyoto Encyclopedia of Genes and Genomes (KEGG) pathway enrichment analysis were performed using the clusterProfiler package in R (v4.14.6). Because goose gene annotation is limited, goose genes were first mapped to their corresponding human orthologs using a three-tier strategy, and enrichment analysis was subsequently conducted based on the mapped gene set. We first obtained explicit human orthologs from NCBI Gene Orthologs through Ensembl Genes release 116 cross-references for GooseV1.0 (GCA_002166845.1) and human GRCh38. We then compared genes without an NCBI mapping bidirectionally using Ensembl release 116 protein sequences and DIAMOND v2.2.4 in very-sensitive mode. We retained only unique reciprocal best hits with an E-value ≤ 1 × 10^−5^, amino-acid identity ≥ 30%, and query and subject coverage ≥ 50%. We mapped the remaining genes by case-insensitive exact matching of Swiss-Prot annotation names to unique human Ensembl gene symbols. We excluded ambiguous one-to-many matches and collapsed repeated human targets from many-to-one mappings before enrichment analysis. This procedure mapped 13,969 of the 14,774 tested genes (94.55%) and 360 of the 383 DEGs (93.99%) to human gene identifiers. The GO and KEGG analyses used the complete human annotation databases as the default backgrounds, comprising 18,723 GO-annotated genes and 9,396 KEGG-annotated genes, respectively. This standardized reference allowed the query and background genes to be evaluated within the same human annotation space and reduced reliance on incomplete goose-specific annotations. *p* values were adjusted using the Benjamini–Hochberg method, and an adjusted *p* value < 0.05 was considered statistically significant.

### 2.8 Gene set enrichment analysis

Gene set enrichment analysis (GSEA) was performed using the clusterProfiler package in R (v4.14.6). Because the gene sets in the Molecular Signatures Database (MSigDB, v2024.1) are mainly based on human gene annotations, goose genes were first mapped to their corresponding human orthologs. All mapped genes were ranked in descending order based on their log₂ fold change values, and GSEA was performed using KEGG pathway gene sets obtained from MSigDB. *p* values were adjusted using the Benjamini–Hochberg method, and a false discovery rate (FDR) < 0.05 was used as the threshold for significant enrichment.

### 2.9 Protein–protein interaction network analysis

Antiviral response- and RIG-I-like receptor signaling-related differentially expressed genes were selected based on the functional enrichment results, mapped to their corresponding human orthologs, and submitted to the STRING database (v12.0) for construction of a protein–protein interaction (PPI) network. Because this analysis was intended for exploratory module detection and candidate-gene prioritization, interactions with a combined score > 0.4 (medium confidence) were retained to balance interaction confidence and network coverage. The resulting network was visualized using Cytoscape (v3.10.4). Candidate hub genes were prioritized using the cytoHubba plugin based on the maximal clique centrality (MCC) algorithm, and network modules were detected using the MCODE plugin. The expression levels of candidate hub genes were represented by FPKM values derived from the RNA-sequencing data, and representative antiviral genes were selected for subsequent RT-qPCR validation of their expression patterns. Glyceraldehyde-3-phosphate dehydrogenase (GAPDH) was used as an internal control, and the primer sequences are shown in Table 1.

**Table 1:** Primer sequences for RT-qPCR.

Name	Sequences (5′ → 3′)	GenBank No.
DDX58	F: AGGAGAGCAGGATATGTAGA R: TCGGTCAGGTAGGATAGAG	XM_048051753.2
IFIH1	F: AATCTGGATAGTGGTTCTGTC R: TATCTTCTGTAGCGGCATCT	XM_013171142.3
DHX58	F: GCTGGTGATAGACGAGTGCC R: TGCGCTGGAGGTAATTCAACA	XM_048053209.2
TRIM25	F: GCTCATAACAAAGTGCTTCT R: CAGTAGTGGATGCCTCTC	XM_048064253.2
RSAD2	F: AGAGCCTTCCAACTCTATCCT R: ACTCCACACATACTTTCCTCC	XM_013172803.3
OASL	F: GGACATCAAGAACCAGACGAT R: GTGCAGAAGCAGGAAGATAGT	XM_048074148.2
GAPDH	F: GCCGAATATGTTGTGGAGTC R: GGAGATGATGACACGCTTAG	XM_067004670.1

### 2.10 RT-qPCR validation

To evaluate the reproducibility of the RNA-seq expression patterns in non-overlapping biological samples, three spleen samples per group that were not included in the RNA-seq analysis were used for RT-qPCR validation. Total RNA was extracted using FreeZol Reagent (R711-02, Vazyme Biotech Co., Ltd., Nanjing, China), and first-strand cDNA was synthesized using HiScript III Reverse Transcriptase (R312-02, Vazyme Biotech Co., Ltd., Nanjing, China) according to the manufacturer’s instructions. RT-qPCR was performed using ChamQ Blue Universal SYBR qPCR Master Mix (Q312-02, Vazyme Biotech Co., Ltd., Nanjing, China) on a CFX Connect Real-Time PCR Detection System (Bio-Rad Laboratories, Inc., Hercules, CA, USA). The thermal cycling program consisted of an initial denaturation at 95 °C for 30 s, followed by 40 cycles at 95 °C for 10 s and 60 °C for 30 s, with a subsequent melting-curve analysis. Each biological sample was analyzed in technical triplicate. GAPDH was used as the reference gene, and relative gene expression was calculated using the 2^−ΔΔCt^ method, with the control group used as the calibrator. Primer sequences are listed in Table 1.

### 2.11 Statistical analysis

All statistical analyses and data visualization were performed using GraphPad Prism 9.5, except for RNA-seq differential expression analysis, enrichment analysis, and PPI network analysis. Data are presented as mean ± standard deviation (SD). Spleen weights and RT-qPCR data were compared between the control and GAstV groups using two-tailed unpaired Student’s t-tests. FPKM expression levels were compared using two-tailed unpaired Mann–Whitney U tests. A *p* value < 0.05, as appropriate, was considered statistically significant.

## 3 Results

### 3.1 Clinical status of goslings and characteristics of spleen samples after GAstV infection

**Figure 1:**
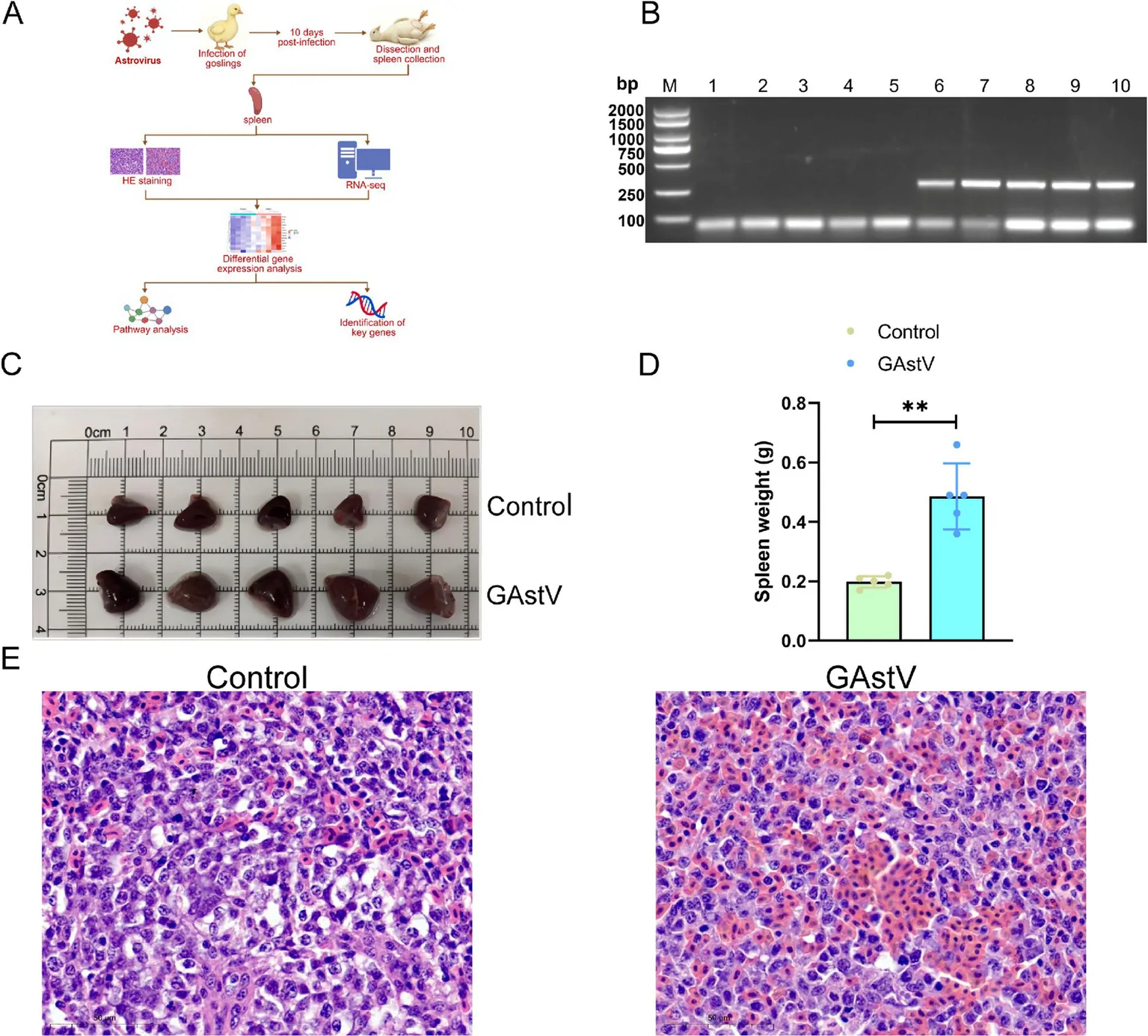
Experimental workflow and characterization of spleen samples after GAstV infection: **(A)** Schematic overview of GAstV infection and subsequent analyses. **(B)** RT-PCR detection of GAstV in spleen tissues. **(C)** Gross morphology of spleens from control and GAstV-infected goslings. **(D)** Spleen weight comparison between control and GAstV-infected groups. **(E)** Representative H&E-stained spleen sections from control and GAstV-infected goslings. Spleen weight data in panel D are presented as mean ± SD (*n* = 5 biological replicates per group). **p < 0.01.

To clarify the experimental design and analytical strategy used to evaluate spleen involvement following GAstV infection, the overall workflow of this study is presented in Figure 1A. At 10 days post-infection, RT-PCR detection showed a GAstV-specific band in spleen tissues from the infected group, whereas no positive signal was observed in the control group, confirming viral presence in the spleen after GAstV infection (Figure 1B). Gross examination revealed that spleens from GAstV-infected goslings were visibly enlarged compared with those from control goslings (Figure 1C). Consistently, spleen weight was significantly increased in the infected group (Figure 1D), indicating marked splenomegaly after infection. H&E staining further showed evident splenic lesions in the infected group, including disorganized tissue architecture, congestion, cellular swelling, vacuolar degeneration, and inflammatory cell infiltration, whereas the control spleens showed relatively normal histological structure (Figure 1E). Together, these findings confirmed successful GAstV infection in the challenged goslings and demonstrated obvious spleen involvement and pathological injury, supporting the use of these spleen samples for subsequent transcriptomic analysis.

### 3.2 Quality assessment of RNA-seq data

**Figure 2:**
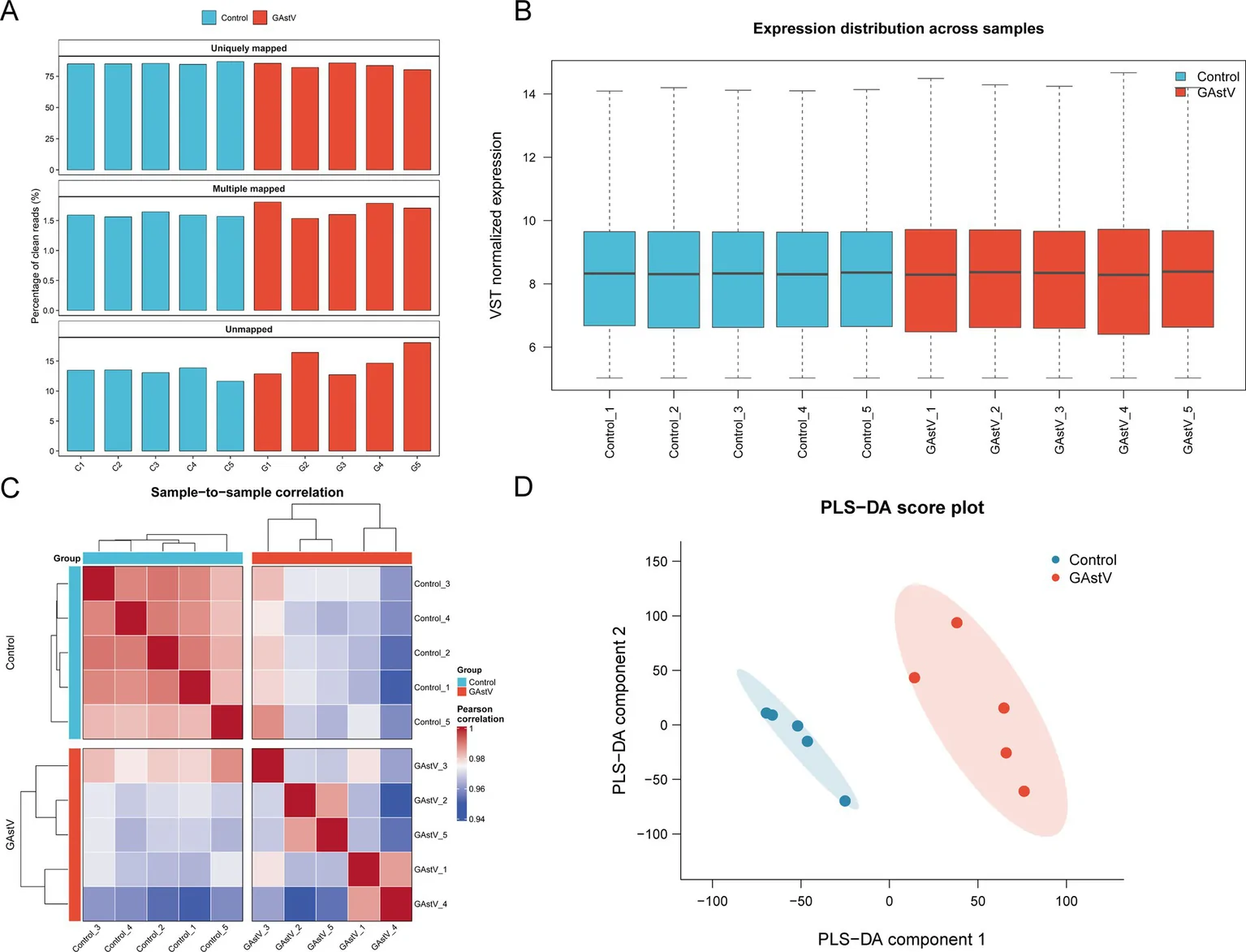
Quality assessment and sample distribution of RNA-seq data from spleens of control and GAstV-infected goslings. **(A)** Read mapping statistics for each sample after alignment to the goose reference genome. **(B)** Boxplot of VST-normalized gene expression values across samples. **(C)** Sample-to-sample correlation heatmap. **(D)** Partial least squares discriminant analysis (PLS-DA) score plot of RNA-seq samples.

To assess RNA-seq data quality, all spleen samples underwent quality control. All samples yielded sufficient clean reads, with high Q20 and Q30 values and GC contents within the expected range (Supplementary Table S1). After alignment to the goose reference genome, all samples showed high overall mapping rates. Uniquely mapped reads accounted for most alignments, whereas the proportions of multi-mapped and unmapped reads were low (Figure 2A). These metrics indicated satisfactory sequencing and alignment quality. Boxplots of VST-transformed expression values showed comparable distributions across samples, supporting consistent normalization among libraries (Figure 2B). Sample-to-sample correlation analysis showed tight clustering of control samples and greater variability among GAstV-infected samples (Figure 2C). Partial least squares discriminant analysis (PLS-DA) further showed group-level separation (Supplementary Table S2). Components 1 and 2 explained 23.67% and 18.99% of the X variance, respectively (Figure 2D). Leave-one-out cross-validation yielded 90% classification accuracy under both distance rules. Exact permutation testing across all 252 balanced label assignments showed that the observed accuracy exceeded chance (max-distance, *p* = 0.0317; centroid-distance, *p* = 0.0476). Nevertheless, the GAstV-infected samples remained more broadly distributed than the control samples (Figure 2D). Collectively, these analyses confirmed the reliability of the RNA-seq data and revealed distinct splenic transcriptomic profiles between control and GAstV-infected goslings.

### 3.3 Identification of differentially expressed genes in the spleen following GAstV infection

**Figure 3:**
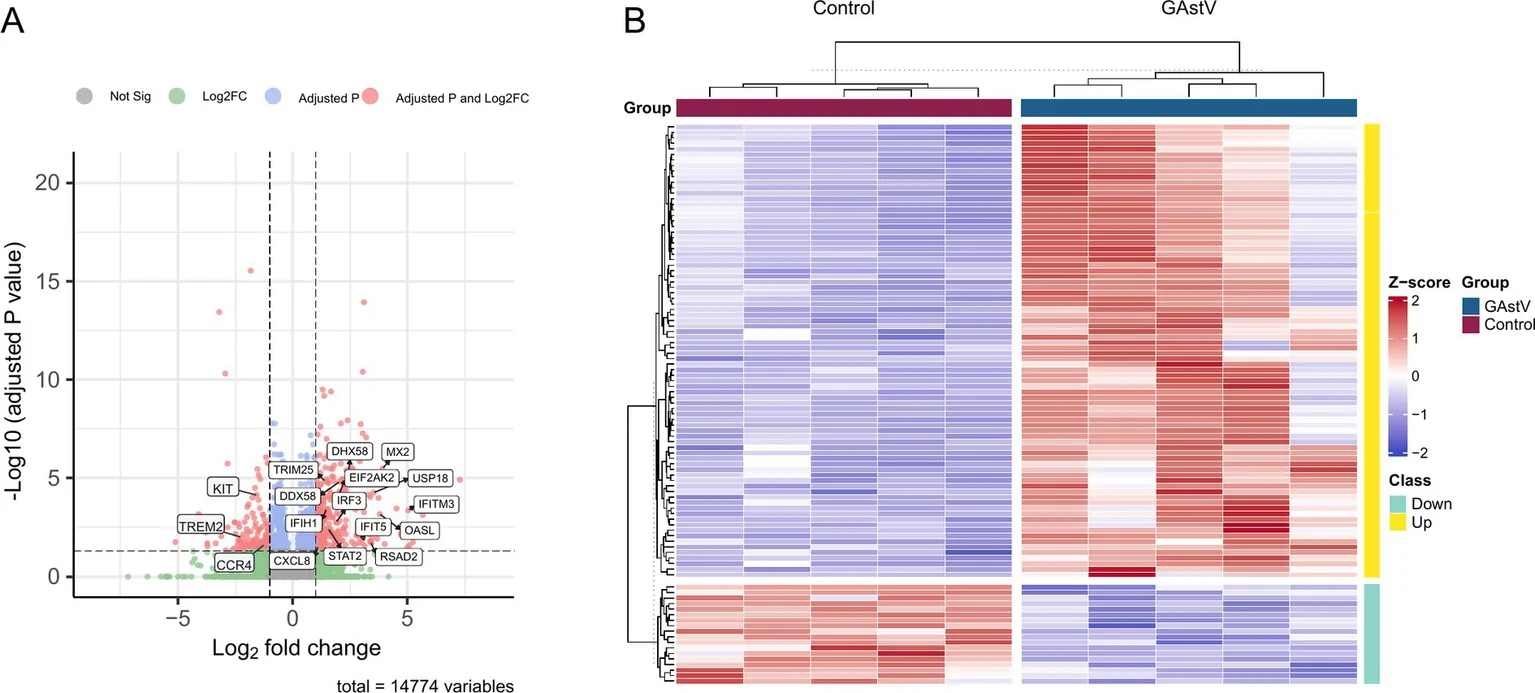
Identification of differentially expressed genes in the spleens of control and GAstV-infected goslings: **(A)** Volcano plot showing the distribution of differentially expressed genes between the control and GAstV-infected groups. **(B)** Hierarchical clustering heatmap of differentially expressed genes in spleen samples.

Differential expression analysis was performed to identify transcriptional changes in the spleen following GAstV infection. A total of 383 differentially expressed genes (DEGs) were identified between the infected and control groups, including 243 upregulated and 140 downregulated genes (Supplementary Tables S3, S4). The volcano plot showed the overall distribution of DEGs, with several antiviral and immune-related genes, such as *IFIH1* (log2FC = 1.48, adjusted *p* = 3.70 × 10^−4^), *DDX58* (log2FC = 2.05, adjusted *p* = 1.54 × 10^−5^), *DHX58* (log2FC = 2.42, adjusted *p* = 8.09 × 10^−6^), *IRF3* (log2FC = 1.89, adjusted *p* = 1.76 × 10^−3^), *TRIM25* (log2FC = 1.40, adjusted *p* = 1.45 × 10^−5^), *STAT2* (log2FC = 1.55, adjusted *p* = 3.75 × 10^−3^), *EIF2AK2* (log2FC = 1.82, adjusted *p* = 2.56 × 10^−4^), *RSAD2* (log2FC = 3.40, adjusted *p* = 0.0170), *OASL* (log2FC = 3.79, adjusted *p* = 6.46 × 10^−4^), *IFIT5* (log2FC = 3.05, adjusted *p* = 1.07 × 10^−2^), *IFITM3* (log2FC = 5.04, adjusted *p* = 4.26 × 10^−4^), *USP18* (log2FC = 3.53, adjusted *p* = 5.42 × 10^−5^), *MX2* (log2FC = 3.92, adjusted *p* = 3.25 × 10^−6^) and *CXCL8* (log2FC = 1.11, adjusted *p* = 2.93 × 10^−2^), prominently upregulated in the infected group (Figure 3A). In contrast, representative downregulated genes included *KIT* (log2FC = −1.57, adjusted *p* = 7.17 × 10^−5^), *TREM2* (log2FC = −2.23, adjusted *p* = 1.04 × 10^−2^), and *CCR4* (log2FC = −1.19, adjusted *p* = 3.14 × 10^−2^). The functional annotations of these genes included immune-cell differentiation, myeloid-cell activation and phagocytosis, and chemotactic responses, respectively. Hierarchical clustering analysis based on all DEGs clearly separated GAstV-infected samples from control samples, indicating distinct splenic transcriptomic profiles between the two groups (Figure 3B).

### 3.4 GO enrichment analysis of differentially expressed genes

**Figure 4:**
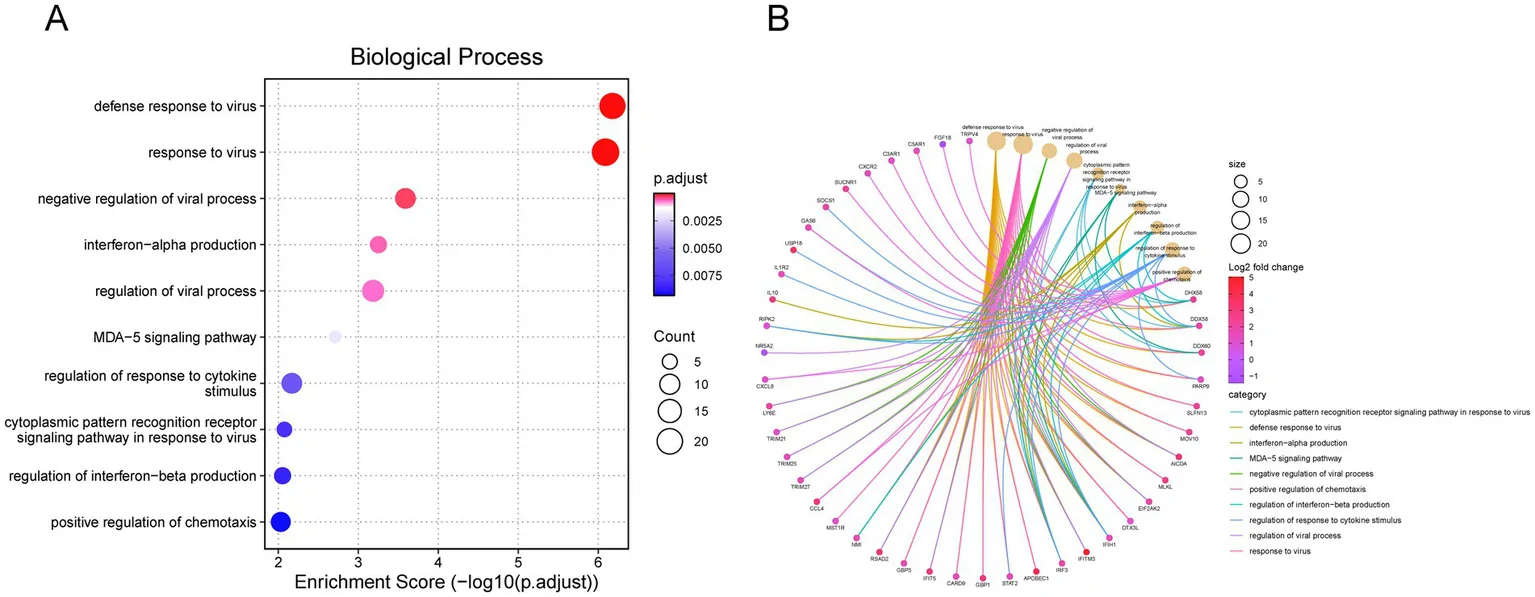
GO biological process enrichment analysis of differentially expressed genes in the spleens of control and GAstV-infected goslings **(A)** Bubble plot of significantly enriched biological process terms. **(B)** Gene-concept network plot showing the relationships between differentially expressed genes and enriched biological process terms.

GO enrichment analysis showed that these DEGs were mainly enriched in biological processes associated with antiviral response, interferon production, and innate immunity (Supplementary Table S5). Among these, defense response to virus and response to virus were the most significantly enriched terms, along with terms related to regulation of viral process, MDA5 signaling pathway, and interferon production (Figure 4A). The cnetplot analysis showed that *IFIH1*, *DHX58*, *DDX58*, *IRF3*, *STAT2*, *IFIT5*, *IFITM3*, *GBP1*, *GBP3*, and *RSAD2* were shared across multiple antiviral-related enriched terms, indicating that these genes were broadly associated with virus recognition and interferon-related processes (Figure 4B).

### 3.5 KEGG pathway enrichment analysis of differentially expressed genes

**Figure 5:**
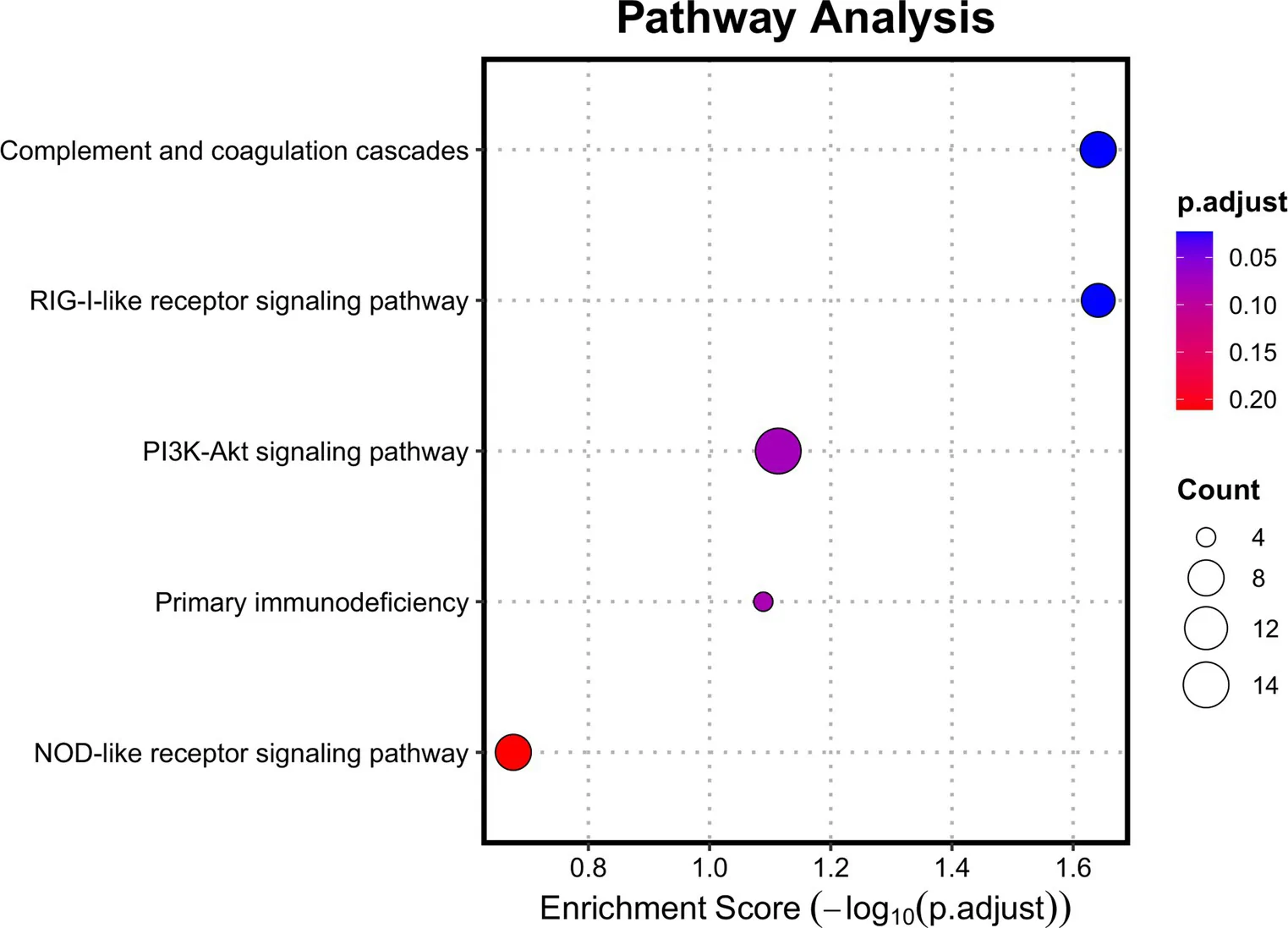
KEGG pathway enrichment analysis of differentially expressed genes.

KEGG enrichment analysis further showed that splenic DEGs following GAstV infection were mainly involved in multiple immune-related pathways (Supplementary Table S6). Specifically, the DEGs were significantly enriched in the complement and coagulation cascades and the RIG-I-like receptor signaling pathway. In addition, the NOD-like receptor signaling pathway and the PI3K–Akt signaling pathway were identified among the enriched pathways (Figure 5). Among these pathways, the RIG-I-like receptor signaling pathway was one of the most prominent antiviral pathways, and was therefore selected for further analysis.

### 3.6 GSEA enrichment analysis

**Figure 6:**
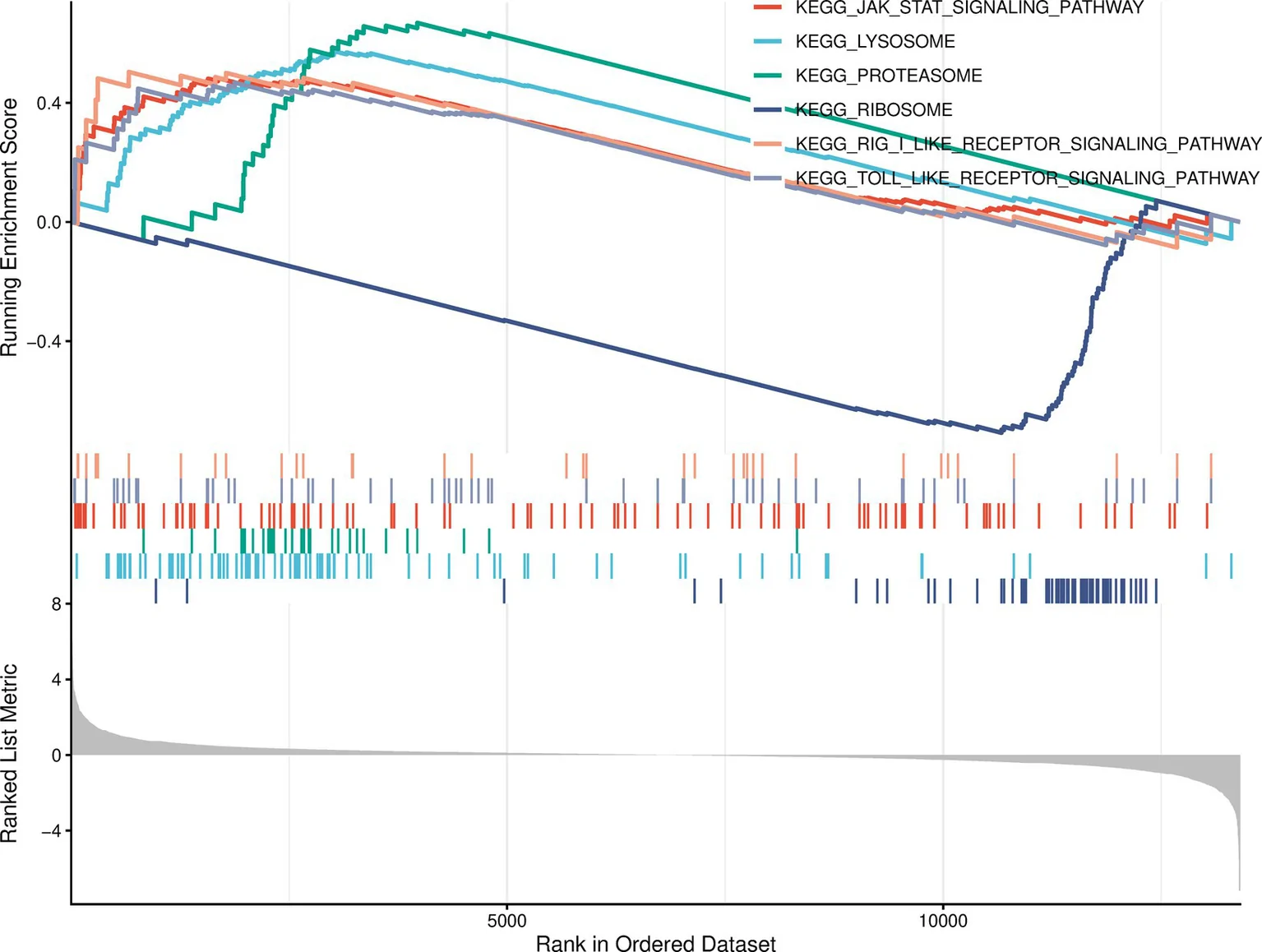
Gene set enrichment analysis of KEGG pathways in spleen transcriptomes from control and GAstV-infected goslings.

To further evaluate whether immune-related pathways showed coordinated changes at the whole-transcriptome level, GSEA was performed using the ranked gene list of all detected genes. KEGG-based GSEA identified several pathways with coordinated enrichment patterns between the GAstV-infected and control groups (Supplementary Table S7), including JAK–STAT signaling pathway, lysosome, proteasome, ribosome, RIG-I-like receptor signaling pathway, and Toll-like receptor signaling pathway (Figure 6). Among these pathways, the RIG-I-like receptor signaling pathway showed a positive enrichment tendency toward the GAstV-infected group. These results were consistent with the DEG-based KEGG enrichment analysis and suggested that RIG-I-like receptor-mediated viral RNA sensing may contribute to the splenic antiviral response following GAstV infection.

### 3.7 Identification of hub genes associated with RIG-I-like receptor-mediated antiviral responses

**Figure 7:**
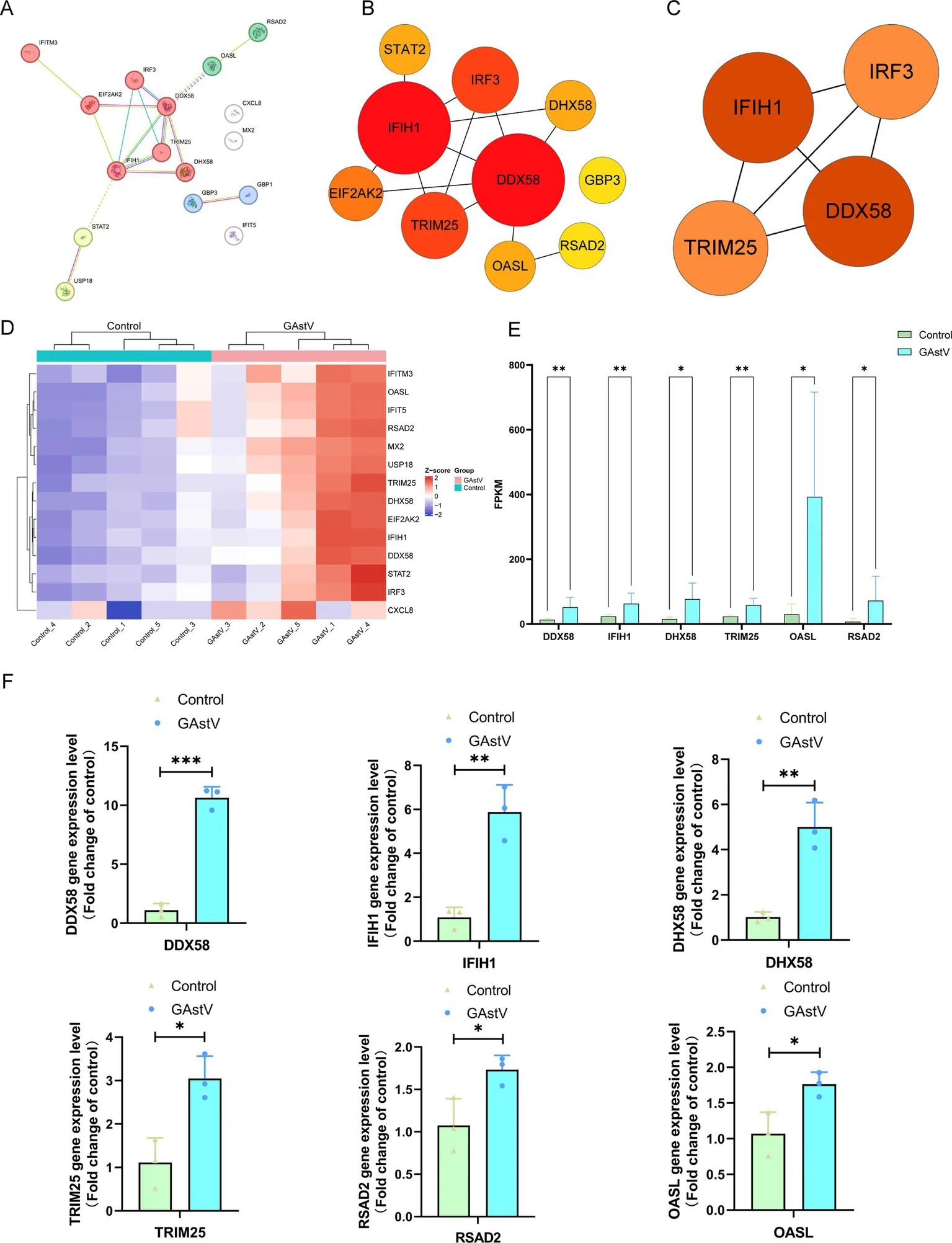
PPI network analysis of selected antiviral response-related differentially expressed genes in the spleens of control and GAstV-infected goslings. **(A)** PPI network of selected antiviral response-related differentially expressed genes. **(B)** Visualization of the top 10 hub genes identified by cytoHubba. **(C)** Key module identified from the PPI network by MCODE analysis. **(D)** Heatmap of selected hub genes. **(E)** FPKM values of selected hub genes in control and GAstV-infected goslings. **(F)** Relative expression levels of selected antiviral genes in control and GAstV-infected goslings. **(A–E)** were based on RNA-seq data from five biological samples per group. Panel F included three independent biological samples per group that were not used for RNA-seq, with each sample analyzed in technical triplicate. Data in panels E and F are presented as mean ± SD. *p < 0.05, **p < 0.01, and ***p < 0.001.

To identify key genes involved in the antiviral response to GAstV infection, a PPI network was constructed using immune- and antiviral-related DEGs (Figure 7A). The MCC algorithm identified *IFIH1*, *DDX58*, *IRF3*, *TRIM25*, *EIF2AK2*, *STAT2*, *DHX58*, *OASL*, *GBP3*, and *RSAD2* as the top 10 hub genes (Figure 7B). In addition, MCODE analysis extracted a highly connected module consisting of *IFIH1*, *DDX58*, *IRF3*, and *TRIM25* (Figure 7C), suggesting that these genes may represent a core antiviral regulatory cluster.

The heatmap showed that most hub genes were upregulated in GAstV-infected spleens compared with controls (Figure 7D). Consistently, FPKM analysis revealed significantly increased expression of several RIG-I-like receptor signaling-related genes, including *DDX58*, *IFIH1*, *DHX58*, *TRIM25*, *RSAD2* and *OASL*, in the infected group (Figure 7E). To further validate the transcriptomic results, RT-qPCR was performed for selected antiviral genes. The expression levels of *DDX58*, *IFIH1*, *DHX58*, *TRIM25*, *RSAD2*, and *OASL* were all significantly elevated after GAstV infection (Figure 7F). These results indicate that GAstV infection activates a RIG-I-like receptor-associated antiviral response in the spleen, with *DDX58*, *IFIH1*, *IRF3*, and *TRIM25* potentially serving as key regulatory nodes.

## 4 Discussion

Previous studies on GAstV have mainly focused on gout, renal injury, and metabolic disorders (19–23), whereas the splenic immune response to GAstV infection remains relatively less characterized compared with renal lesions and gout-associated pathology (8). In the present study, infected goslings showed obvious splenomegaly, positive viral detection in the spleen, and histopathological lesions, including tissue disorganization, congestion, vacuolar degeneration, and inflammatory cell infiltration. These findings indicate that the spleen is involved in GAstV infection and may participate actively in the host response rather than being only a secondary target organ.

KEGG analysis identified the complement and coagulation cascades as a top-ranked pathway, providing a possible link between the splenic response and the systemic lesions of GAstV infection. Eight DEGs contributed to this enrichment. The upregulated genes included *C1QB* (log2FC = 1.65, adjusted *p* = 5.13 × 10^−3^), *C1S* (log2FC = 1.32, adjusted *p* = 2.60 × 10^−2^), and *C3AR1* (log2FC = 1.23, adjusted *p* = 2.26 × 10^−4^). *C5AR1* (log2FC = 1.36, adjusted *p* = 3.89 × 10^−2^) and *SERPING1* (log2FC = 3.10, adjusted *p* = 1.26 × 10^−14^) were also upregulated. In contrast, *F13B* (log2FC = −1.47, adjusted *p* = 2.23 × 10^−2^), *BDKRB2* (log2FC = −2.07, adjusted *p* = 2.24 × 10^−2^), and *CFHR1* (log2FC = −1.34, adjusted *p* = 4.79 × 10^−2^) were downregulated. Monosodium urate crystals can activate complement and generate C3a and C5a, with C5a promoting IL-1 production and leukocyte recruitment (24). The increased expression of *C3AR1* and *C5AR1* may therefore reflect altered responsiveness to complement-derived inflammatory signals associated with urate deposition. Changes in *F13B* and *BDKRB2* also point to altered coagulation and vascular regulation, which may be relevant to the splenic congestion observed here. GAstV infection can also cause hepatic sinus narrowing (22), together with renal tubular injury and urate deposition (19, 23). However, the concurrent upregulation of SERPING1 and downregulation of CFHR1 indicate that the pathway did not change uniformly. These results should therefore be interpreted as transcriptional perturbation of complement- and coagulation-related processes rather than direct evidence of complement activation or coagulopathy. Because the data were obtained from spleen tissue at 10 dpi, it remains unclear whether these changes contribute to urate deposition and renal injury or occur secondarily to systemic inflammation. Further measurements of complement activation products, coagulation parameters, and renal tissue changes are needed to clarify this relationship.

The spleen is a major immune organ involved in systemic immune regulation, and transcriptomic studies in chickens have shown that splenic gene expression changes can reflect immune functional alterations (10, 25). As GAstV is an RNA virus, one of the earliest events after infection is likely the recognition of viral RNA by host pattern-recognition receptors (26, 27). Previous studies have also shown that GAstV or goose-origin nephrotic astrovirus infection can activate host innate immune responses (28). Our data suggest that this process may be mainly associated with the RIG-I-like receptor pathway. In particular, *DDX58*, *IFIH1*, and *DHX58* were all markedly upregulated in infected spleens. *DDX58* encodes RIG-I and *IFIH1* encodes MDA5, both of which are central cytoplasmic sensors of viral RNA, whereas DHX58 acts as an important regulator of RIG-I and MDA5-mediated signaling (27). The concurrent increase in these genes suggests that GAstV infection may activate a coordinated viral RNA-sensing response in the spleen.

Downstream molecules in this pathway were also induced. *TRIM25*, which is important for RIG-I activation (29, 30), and *IRF3*, a key transcription factor involved in interferon induction (26, 27, 31), were both identified as central antiviral genes in the infected spleen. These findings suggest the possible involvement of a RIG-I/MDA5-associated antiviral signaling cascade in splenic tissue. Previous studies reported similar induction of antiviral genes during GAstV infection in other systems (29, 32). Our results extend these observations to the goose spleen and suggest that viral RNA sensing through RIG-I-like receptors may be an important component of the splenic innate immune response to GAstV.

Another notable feature of this response was the upregulation of *STAT2* and multiple interferon-stimulated antiviral genes. These genes are involved in several stages of antiviral defense, such as viral RNA recognition, inhibition of translation, restriction of viral replication, and regulation of interferon signaling (33, 34). Their coordinated induction suggests that GAstV infection may establish an interferon-associated antiviral state in the spleen. Such a response is likely beneficial for limiting viral spread, but it may also reflect strong immune activation within splenic tissue.

This point is important because the pathological changes observed in the spleen suggest that antiviral defense and tissue injury may occur simultaneously. Inflammatory cell infiltration and congestion were evident in infected spleens, and *CXCL8* was also among the upregulated genes. This suggests that GAstV infection may promote leukocyte recruitment and local inflammatory responses in the spleen (35). Therefore, the splenic lesions observed in infected goslings may not be caused only by direct viral injury, but may also be associated with excessive innate immune activation (36, 37). In this context, activation of the RIG-I-like receptor pathway may help restrict GAstV infection while also contributing to inflammatory damage when strongly activated (37).

Previous studies have shown that in the canonical RIG-I-like receptor pathway, viral RNA recognition by RIG-I and MDA5 is followed by signal transduction through MAVS and downstream kinase complexes, including TBK1 and IKK (38). This signaling cascade activates transcription factors such as IRF3 and NF-κB, thereby promoting type I interferon production and inflammatory cytokine expression (39–41). Secreted type I IFNs can subsequently activate IFNAR-mediated JAK–STAT signaling and induce a broad set of interferon-stimulated genes (42–44). In the present study, the upregulation of *DDX58*, *IFIH1*, *DHX58*, *TRIM25*, *IRF3*, *STAT2*, and multiple ISGs is consistent with this previously described antiviral signaling framework. Nevertheless, although MAVS, TBK1, IKK, and NF-κB are well-established components of this pathway, their activation in GAstV-infected goose spleens was not directly examined at the protein or phosphorylation level in the present study. Therefore, the involvement of these canonical signaling intermediates in GAstV-infected goose spleens should be interpreted cautiously and requires further experimental validation in this infection context.

**Figure 8:**
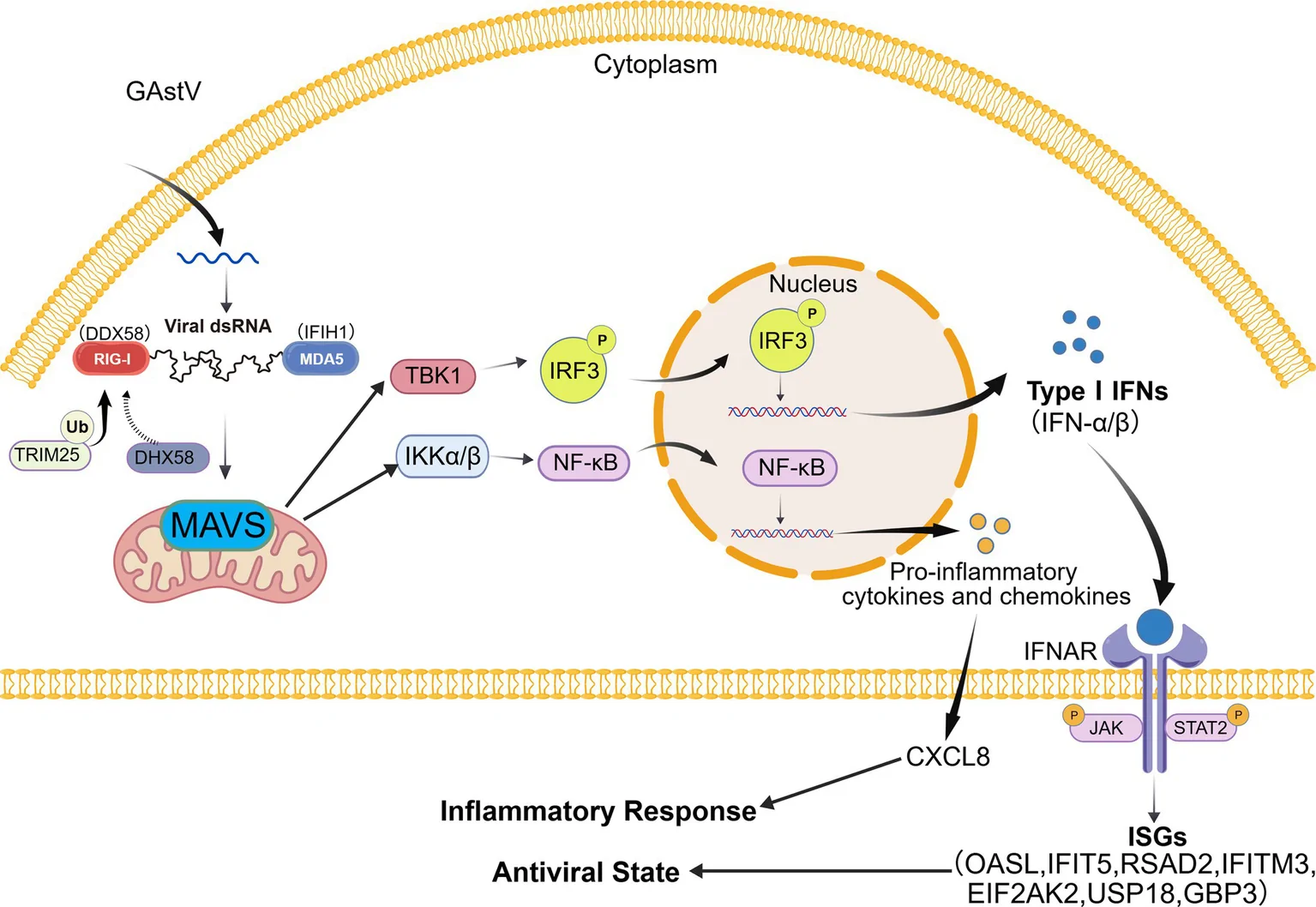
Transcriptome-supported working model of GAstV-associated innate immune responses in gosling spleens at 10 dpi. Transcriptomic data, supported by RT-qPCR validation of selected genes, revealed increased expression of viral RNA-sensing-related genes and multiple interferon-stimulated genes, suggesting the possible involvement of RIG-I-like receptor-related antiviral responses. MAVS/TBK1/IKK signaling, IRF3 and NF-κB activation, type I IFN production, and JAK–STAT signaling are depicted as inferred canonical events that were not directly validated. The temporal and causal relationships among viral sensing, ISG induction, inflammation, and splenic injury remain unresolved.

To integrate these transcriptomic and histopathological findings, we propose a working model for the splenic innate immune response to GAstV infection (Figure 8). In this model, GAstV RNA may be recognized by RIG-I-like receptors, as supported by the upregulation of *DDX58*, *IFIH1*, and *DHX58* in infected spleens. *TRIM25*, which facilitates RIG-I activation, and *IRF3*, a key transcription factor involved in interferon induction, were also increased, suggesting that GAstV infection may activate an antiviral signaling cascade associated with RIG-I and MDA5. Consistent with this model, the marked induction of interferon-stimulated genes further indicates the establishment of an interferon-associated antiviral state in the spleen. In parallel, the upregulation of *CXCL8* and the presence of inflammatory cell infiltration suggest that this antiviral response may be accompanied by chemokine-mediated inflammatory activation. Therefore, the proposed model links GAstV-induced viral RNA sensing with both antiviral defense and inflammatory splenic injury.

Previous studies have reported that astrovirus infection can cause splenic lymphocyte depletion, apoptosis, and immunosuppression in goslings (15, 45). Several genes associated with immune-cell development and function, including *KIT*, *TREM2*, and *CCR4*, were downregulated in infected spleens. Their reduced expression may be consistent with the previously reported splenic lymphocyte depletion and immunosuppression. These findings complement previous reports by showing that infected spleens also mounted an antiviral transcriptional response. At 10 dpi, antiviral activation coexisted with reduced expression of immune-cell-associated genes. Whether these concurrent transcriptional changes contribute to splenic injury and immune dysfunction requires further investigation.

Several limitations should be acknowledged. First, spleen tissues were collected only at 10 dpi; therefore, the present study provides a single-time-point transcriptional snapshot and cannot distinguish early viral RNA-sensing events from sustained antiviral responses or secondary transcriptional changes associated with established inflammation and tissue injury. Second, intraperitoneal inoculation bypasses the predominantly fecal–oral route of GAstV transmission and may elicit splenic responses that differ in magnitude or kinetics from those occurring during natural infection. Third, although RNA-seq and RT-qPCR revealed increased expression of DDX58, IFIH1, DHX58, TRIM25, IRF3, STAT2, and multiple ISGs, these findings provide pathway-level evidence rather than direct functional validation of RIG-I-like receptor signaling. The specific roles of RIG-I and MDA5 in GAstV recognition, as well as downstream events involving MAVS, TBK1, IKK, and IRF3, require further validation at the protein and phosphorylation levels. Fourth, the STRING network topology, MCC-based hub-gene ranking, and MCODE-defined modules may vary with the selected confidence threshold. The identified genes and modules should therefore be regarded as network-prioritized candidates, and the predicted protein interactions require experimental validation. Fifth, because the GO and KEGG analyses relied on mapped human orthologs and complete human annotation backgrounds, conserved and well-annotated genes may be preferentially represented, whereas goose-specific or poorly annotated responses may be underrepresented. Finally, the splenic cell populations responsible for these responses remain unknown. Future studies incorporating infection models that more closely mimic natural exposure, multiple post-infection time points, protein-level and functional validation, quantitative histopathological scoring, and cell-type-specific analyses will be needed to define the temporal and mechanistic relationships among viral sensing, antiviral responses, and splenic injury. Despite these limitations, our findings provide a transcriptomic framework and identify candidate pathways and genes for further investigation of GAstV pathogenesis in goslings.

## 5 Conclusion

GAstV infection caused obvious splenic lesions and markedly altered the splenic transcriptional profile of goslings. The host response was characterized by enhanced antiviral innate immune signaling, particularly enrichment of the RIG-I-like receptor pathway and upregulation of related genes, including *DDX58*, *IFIH1*, *DHX58*, *TRIM25*, and *IRF3*. These findings suggest that RIG-I-like receptor-mediated viral RNA sensing is likely involved in the splenic response to GAstV infection and provide new insight into host innate immune responses associated with GAstV infection.

## Data Availability

The datasets presented in this study can be found in online repositories. The names of the repository/repositories and accession number(s) can be found at: PRJNA1479086 (NCBI-BioProject-SRA).

## Supplementary material

Supplementary Table S1 Summary of RNA-seq sequencing quality metrics for each spleen sample.

Supplementary Table S2 PLS-DA explained variance, cross-validation, and permutation-test results.

Supplementary Table S3 Expression profiles and annotations of all expressed genes.

Supplementary Table S4 Differentially expressed genes between GAstV-infected and control gosling spleens.

Supplementary Table S5 GO enrichment analysis of differentially expressed genes.

Supplementary Table S6 KEGG pathway enrichment analysis of differentially expressed genes.

Supplementary Table S7 GSEA results based on ranked expressed genes.
