# Supplementary material for: Splenic transcriptomic profiling reveals innate immune responses and RIG-I-like receptor signaling in goslings infected with goose astrovirus
Source: Front Vet Sci. 2026 Aug 26;13:1911277. doi: 10.3389/fvets.2026.1911277 (PMC13553421; doi:10.3389/fvets.2026.1911277)
Supplement: Supplementary file 1 [file Data_Sheet_1.zip › 1911277_SupMaterial/Table 1.DOCX]

Table S1 Primer sequences for qRT-PCR

| Name | Sequences (5’→3’) | GenBank No. |
| --- | --- | --- |
| DDX58 | F：AGGAGAGCAGGATATGTAGA  R：TCGGTCAGGTAGGATAGAG | XM_048051753.2 |
| IFIH1 | F：AATCTGGATAGTGGTTCTGTC  R：TATCTTCTGTAGCGGCATCT | XM_013171142.3 |
| DHX58 | F：GCTGGTGATAGACGAGTGCC  R：TGCGCTGGAGGTAATTCAACA | XM_048053209.2 |
| TRIM25 | F：GCTCATAACAAAGTGCTTCT  R：CAGTAGTGGATGCCTCTC | XM_048064253.2 |
| RSAD2 | F：AGAGCCTTCCAACTCTATCCT  R：ACTCCACACATACTTTCCTCC | XM_013172803.3 |
| OASL | F：GGACATCAAGAACCAGACGAT  R：GTGCAGAAGCAGGAAGATAGT | XM_048074148.2 |
| GAPDH | F：GCCGAATATGTTGTGGAGTC  R：GGAGATGATGACACGCTTAG | XM_067004670.1 |
